# Optimizing Smartphone-Delivered Cognitive Behavioral Therapy for Body Dysmorphic Disorder Using Passive Smartphone Data: Initial Insights From an Open Pilot Trial

**DOI:** 10.2196/16350

**Published:** 2020-06-18

**Authors:** Hilary Weingarden, Aleksandar Matic, Roger Garriga Calleja, Jennifer L Greenberg, Oliver Harrison, Sabine Wilhelm

**Affiliations:** 1 Massachusetts General Hospital/Harvard Medical School Boston, MA United States; 2 Telefónica Alpha Barcelona Spain

**Keywords:** body dysmorphic disorder, cognitive behavioral therapy, mobile health, mobile phone, patient engagement

## Abstract

**Background:**

Smartphone-delivered cognitive behavioral therapy (CBT) is becoming more common, but research on the topic remains in its infancy. Little is known about how people typically engage with smartphone CBT or which engagement and mobility patterns may optimize treatment. Passive smartphone data offer a unique opportunity to gain insight into these knowledge gaps.

**Objective:**

This study aimed to examine passive smartphone data across a pilot course of smartphone CBT for body dysmorphic disorder (BDD), a psychiatric illness characterized by a preoccupation with a perceived defect in physical appearance, to inform hypothesis generation and the design of subsequent, larger trials.

**Methods:**

A total of 10 adults with primary diagnoses of BDD were recruited nationally and completed telehealth clinician assessments with a reliable evaluator. In a 12-week open pilot trial of smartphone CBT, we initially characterized natural patterns of engagement with the treatment and tested how engagement and mobility patterns across treatment corresponded with treatment response.

**Results:**

Most participants interacted briefly and frequently with smartphone-delivered treatment. More frequent app usage (*r*=–0.57), as opposed to greater usage duration (*r*=–0.084), correlated strongly with response. GPS-detected time at home, a potential digital marker of avoidance, decreased across treatment and correlated moderately with BDD severity (*r*=0.49).

**Conclusions:**

The sample was small in this pilot study; thus, results should be used to inform the hypotheses and design of subsequent trials. The results provide initial evidence that frequent (even if brief) practice of CBT skills may optimize response to smartphone CBT and that mobility patterns may serve as useful passive markers of symptom severity. This is one of the first studies to examine the value that passively collected sensor data may contribute to understanding and optimizing users’ response to smartphone CBT. With further validation, the results can inform how to enhance smartphone CBT design.

## Introduction

### Background

The supply and demand imbalance between those who need psychological treatment and those who are able to receive it represents a serious public health concern [1,2]. Indeed, only 43.6% of those with psychiatric illnesses in the United States receive treatment and fewer receive gold-standard treatment [2]. Moreover, certain psychiatric illnesses are less well-recognized than others, and under-recognized illnesses likely have the biggest access to care gaps. For example, 35.1% of adults with body dysmorphic disorder (BDD), a psychiatric illness characterized by a preoccupation with a perceived defect in physical appearance [3], receive psychotherapy; only 17.4% with BDD receive the gold-standard cognitive behavioral therapy (CBT) [4], despite strong research demonstrating its efficacy [5-7].

Fortunately, the development of smartphone-delivered CBT treatments may help address this access gap. Compared with in-person therapy, smartphone-delivered CBT is less expensive, more widely accessible, and highly flexible (eg, it can be used anywhere and anytime patients have their phones). The potential benefits of smartphone-delivered CBT are compounded by the growth of smartphone ownership. At present, 81% of the US population own a smartphone, a rate that has more than doubled since 2011 [8]. Not surprisingly, therefore, there is mounting enthusiasm among clinical researchers for developing and deploying smartphone CBT treatments [9,10].

Despite growing excitement, our understanding of smartphone-delivered CBT remains in its infancy, with a dramatic gap between the number of publicly available mental health apps and the paucity of scientific papers reporting on their evaluation [11]. In particular, very little is known about how people naturally engage with smartphone-delivered CBT compared with traditional in-person treatments, but it is likely that usage patterns differ dramatically. For example, in-person CBT is most commonly administered in once weekly, 50-min sessions, representing a concentrated and infrequent format. Next-generation internet-based CBT (ICBT) treatments, which have garnered substantial empirical support [12,13], are often built to mimic this style of longer duration, spaced out, formalized sessions because they were designed to be completed on one’s home computer. In both traditional CBT and ICBT, patients are instructed to practice skills between sessions to reinforce learning within real-world settings. Whereas the practice of skills between sessions has been associated with better CBT outcomes [14-16], many patients struggle to practice skills on their own between sessions. On the other hand, because people carry their phones at most times, smartphone-delivered treatments can be accessed by users at nearly any time and place. Having smartphone-delivered support available at all times may encourage practicing CBT skills with greater frequency and in a wider variety of settings than traditional in-person CBT, potentially opening doors to highly distinct engagement patterns. However, to date, we know very little about how often, for how long, or where people naturally engage with smartphone-delivered CBT treatments.

Moreover, very little is known about which engagement patterns correspond with an optimal response to smartphone-delivered CBT. Understanding optimal engagement patterns can allow for the design of more potent treatments by seeking to promote the most effective patterns of CBT app use. For example, gaining information about whether one’s frequency of use or duration of use matters more in terms of treatment response can inform whether apps should be designed to promote bursts of brief engagement or longer, less frequent sessions.

Finally, little is currently known about how the mobility patterns of patients change over the course of smartphone-delivered CBT. Previous research suggests that time spent at home, measured via a GPS, can serve as a digital marker of avoidance [17] and may correlate with symptom severity in depressive disorders [18]. Therefore, obtaining initial information about how mobility patterns change across smartphone treatment, and how these changes correspond with changes in severity, can inform treatment optimization by passively detecting changes in severity and triggering just-in-time interventions.

Altogether, in the field’s current, early stage of developing smartphone-delivered CBT treatments, we can benefit from examining pilot engagement and mobility data, to shape how we design optimal digital services and their clinical trials in the future. Smartphones offer a unique avenue for gaining rich insights into patterns of treatment engagement and predictors of treatment response because smartphones can unobtrusively (ie, in the background, without user input) collect a wide variety of sensor-based data over the course of treatment. For example, with patient consent, smartphones can be configured to passively collect objective information about patients’ engagement with the app (ie, how often and for how long patients use the program) as well as patients’ behavioral patterns over the course of treatment (eg, where patients typically use the app, changes in mobility patterns across treatment, via GPS). Passive data offer notable strengths for learning how to optimize smartphone-delivered treatments compared with more traditional assessment methods such as clinician interviews and self-reports. Passive smartphone data are sampled at a far greater frequency than traditional clinical assessments, which, at most, might be administered weekly. Frequent assessment that is conducted as one lives daily life captures richer contextual information, has higher temporal resolution to detect changes in symptoms or severity, and reduces the influence of recall biases that arise from subjective recollection of experiences over a broad time frame [19]. Altogether, passive smartphone data can offer valuable, low-burden insights into patterns of treatment engagement and digital markers of progress or deterioration, to optimize future design and research of smartphone-delivered treatments [20].

### Objectives

To this end, this study exploratorily examines passive smartphone data from a 12-week open pilot trial of a smartphone-delivered CBT (*Perspectives*) for patients with BDD (N=10) to inform the study design, variables of interest, and hypothesis generation for future trials of smartphone-delivered CBT services. First, we aimed to initially characterize typical patterns of engagement with smartphone-delivered CBT for BDD in our sample, to obtain a preliminary understanding of how engagement may be similar to or different from participation in traditional in-person CBT. Second, we aimed to initially test how patterns of engagement corresponded with treatment response to inform early hypotheses about how we may design apps to optimize engagement and response. Third, we aimed to initially characterize the mobility patterns of participants across treatment, to preliminarily test whether GPS-based mobility patterns could serve as a digital marker of disorder severity. If validated in larger trials, digital markers of severity could be used to enhance treatments by triggering just-in-time interventions.

## Methods

### Participants and Recruitment

A paper by Wilhelm et al [21] gives detailed information on study methods, including a Consolidated Standards of Reporting Trials diagram, participant demographic information, and a description of the smartphone-delivered CBT for BDD treatment (ClinicalTrials.gov Identifier: NCT03221738).

A total of 10 adults with a primary psychiatric diagnosis of BDD were enrolled nationally in the open pilot trial (female: n=8, male: n=2; mean age 27.6, SD 5.66 years). Other inclusion criteria required that participants had at least moderately severe BDD symptoms (defined as a Yale-Brown obsessive compulsive scale modified for BDD [BDD-YBOCS] score >20), an acuity level appropriate for an outpatient level of care and lived in the United States. Exclusion criteria prohibited participation if the individual had a current severe major depressive disorder; borderline personality disorder; substance use disorder or acute, active suicidal ideation; had a lifetime diagnosis of bipolar disorder or a psychotic disorder; had cognitive impairment or intellectual disability that would interfere with participation; had engaged in previous CBT for BDD, or did not own an iPhone that supported the app software. Participants were either unmedicated or those on medication were required to be on a stable dose for at least two months before starting the study and were instructed not to change their medication regimen during the trial.

### Procedures

Procedures were approved by the hospital’s institutional review board, and participants provided informed consent before beginning the study. Informed consent included a description of each type of passive smartphone data to be collected, a description of how those data were securely transmitted and deidentified before storage, the rationale for collecting those data, and a description of who would have access to the data.

#### Assessments

Clinical assessments were conducted by reliable, independent evaluators with a Master’s degree or doctorate, who were trained in primary diagnostic and outcome measures. Assessments for this study were conducted at the screening and baseline (same visit; week 0), midpoint (week 6), and posttreatment (week 12) assessments, and participants were compensated US $25 for completing the week 6 and week 12 assessments. Clinician-administered measures were collected via secure video calls that were Health Insurance Portability and Accountability Act (HIPAA) compliant. Self-report data were collected via Research Electronic Data Capture [22], a secure, HIPAA-compliant web-based survey collection platform.

In addition to providing clinical and outcome data, participants also provided qualitative feedback on the CBT app at several time points across the study. Specifically, written feedback was collected at the posttreatment assessment; oral feedback was gathered by members of the design team via separate interviews conducted shortly after the baseline, midpoint, and posttreatment clinical assessments.

#### Treatment

Following the screening and baseline assessment, the study staff instructed eligible participants on how to download and activate the *Perspectives* app onto their personal smartphones. The 12-week treatment consisted of psychoeducation and self-paced interactive exercises presented in a fixed order, which taught each of the core CBT skills for BDD (ie, cognitive restructuring, exposure with ritual prevention, mindfulness and perceptual retraining, core beliefs and self-esteem, engagement in value-based activities, and relapse prevention). The treatment was delivered via the smartphone app and was supported by light-touch communication with a doctoral-level therapist, whose primary role was to enhance motivation, address roadblocks, and answer questions [21]. Note that in this trial, *Perspectives* was developed for iPhones only; in 2018, iPhone operating systems represented approximately 44% of smartphones in the United States [23].

#### Passive Smartphone Data Collection

*Perspectives* was configured to passively collect information about app usage and mobility patterns of participants via GPS (the Measures section gives further details). We chose to collect these 2 types of passive data based on previous literature that points to their utility. In particular, app usage data may offer valuable insights into which engagement patterns are optimal for promoting treatment response [20], whereas mobility patterns from GPS can detect the proportion of time spent at home, a potential digital marker of avoidance [17]. As BDD is characterized by substantial avoidance (including housebound avoidance) [24], mobility patterns, therefore, have the potential to passively detect signs of symptom severity. By carefully selecting data categories and sampling rates (by default, the location was sampled whenever location changed by at least 100 m), the app was optimized to balance battery life and allowance of natural phone use. To this end, no participants complained about battery problems during the study.

### Measures

#### Clinical Assessments

The Mini-International Neuropsychiatric Interview (version 7.0.2) [25] is a semistructured, clinician-administered diagnostic assessment of psychiatric illnesses. It was administered at the screening assessment to evaluate the inclusion and exclusion criteria.

The BDD-YBOCS [26] is a semistructured, clinician-administered, gold-standard assessment of current BDD symptom severity. The BDD-YBOCS is a 12-item Likert scale. Total scores range from 0 to 48, with higher scores corresponding to greater BDD severity. The BDD-YBOCS has strong psychometric properties, including internal consistency, interrater reliability, and test-retest reliability [26,27]. The BDD-YBOCS was administered at each assessment to evaluate the eligibility criteria (at screening) and changes in BDD severity. Percentage improvement in severity, a primary outcome in this study, was computed by dividing the difference between baseline and posttreatment (week 12) BDD-YBOCS scores by the baseline value.

#### Passive Smartphone Features

To quantify and analyze the patterns of engagement with *Perspectives* and mobility across treatment, we computed several variables based on passive smartphone data.

##### Quantity of App Use

The quantity of app use was calculated as the total duration in minutes that a participant used the app. This was calculated by adding together all app *sessions*, or the periods of on-app time devoted to the therapy. Before analyses, together with designers of the *Perspectives* app, we considered various cutoff points for outliers in session length. Taking into account the possibility that participants might occasionally engage in multiple longer components of the app in sequence (eg, a mindfulness audio exercise, responding to coach messages, and completing an exposure exercise), we decided *a priori* on a session length cutoff of approximately 60 min, and outliers beyond this length were removed. To account for *bursty* usage (ie, multiple brief usages separated by short breaks of <60 min in between), app usages that were separated by <60 min were summed together into a single *session*. For example, a participant who used the app for two 10-min increments with a 5-min break in between would be logged as having one 20-min session during this span. Quantity of app use was computed for the first half (6 weeks) and for the full 12 weeks of the CBT program (Table 1).

##### Frequency of App Use

This metric measured the extent to which a participant tended to use the app frequently or infrequently, expressed as the mean duration between 2 consecutive sessions, or periods of uninterrupted use. Frequency of app use was computed for the first half (6 weeks) and the full 12 weeks of the CBT program (Table 1).

##### Mobility Patterns

Using GPS data, we calculated the percentage of time spent at home during 1-week time intervals that overlapped with baseline, midpoint, and posttreatment BDD-YBOCS assessments (including 3 days before, 3 days after, and the day of BDD-YBOCS administration). Of note, at baseline, the BDD-YBOCS was typically administered on the same day the app was installed. Therefore, GPS data were not generally available for the 3 days before the baseline BDD-YBOCS assessment. Home location was inferred as the most common location ID captured between 3 AM and 6 AM per individual. All the remaining location IDs were labeled as *outside of home*. The various locations of participants were collected in a privacy-preserving way; each location where a participant spent at least 30 min was assigned a unique and random location ID (eg, *ID78*) and stored in the logs. This procedure was performed locally on the phone, and raw locations were removed before transferring the data to the server. The GPS sampling rate was set to 15 min, yet GPS readings were missing for 60% of the days.

### Statistical Analyses

Data were analyzed using Python 3.6 (Python Software Foundation).

#### Descriptive Patterns of App Usage

To characterize the overall patterns of app usage, we visually inspected longitudinal patterns of usage by the participants across the 12-week treatment and we calculated the number of times the participants were engaged with the app for different lengths of time (ie, session durations). We elected not to identify subsamples based on usage (ie, clusters of users with similar engagement patterns) either visually or quantitatively, because of the small sample size.

#### App Usage Patterns as Correlates of Percentage Improvement in the Yale-Brown Obsessive Compulsive Scale Modified for Body Dysmorphic Disorder

To examine how the patterns of engagement of participants with *Perspectives* corresponded with their percentage improvement in BDD severity, we focused on 2 types of app usage patterns: quantity of app usage and frequency of app usage across the treatment. Normality was tested using the Shapiro-Wilk test and visual inspection. As the frequency of app use variable followed a long-tail distribution, log-transformation was performed before the analysis.

Two bivariate correlations were conducted, to preliminarily explore the relationships between the variables measuring (a) quantity and (b) frequency of app usage with percentage improvement in BDD-YBOCS from the baseline to week 12. Next, to initially examine the relative effect of quantity versus frequency of app use, a regression analysis of percent improvement was conducted, with both quantity and frequency of app use as independent variables. We primarily evaluated effect sizes, as opposed to statistical significance, for correlation and regression analyses, given the pilot nature of the data.

#### GPS Data as a Correlate of the Yale-Brown Obsessive Compulsive Scale Modified for Body Dysmorphic Disorder Scores

The relationship between symptom severity and mobility was explored via a bivariate correlation between BDD-YBOCS scores and the percentage of time spent at home during the week the BDD-YBOCS was measured. Note that absolute BDD-YBOCS scores were used for this analysis instead of percentage improvement, given the goal of exploring the predictive power of a GPS marker in assessing the current acuity of participants. The correlation analysis included 30 pairs of location variables and BDD-YBOCS scores (ie, 3 per participant, at baseline, midpoint, and posttreatment); thus, each participant was equally represented in the correlation analysis. Given that this analysis included multiple time points per participant, we followed up with a secondary analysis to verify that the results were not inflated based on the longitudinal nature of the data. Namely, 6000 correlation analyses were run by randomly selecting 1 of the 3 time points per participant (pre-, mid-, or posttreatment). This approach resulted in a very similar median correlation value to the analysis with 3 time points per participant; thus, secondary results are not presented. Again, we primarily evaluated the effect size, as opposed to statistical significance, for this correlation analysis, to best account for the pilot nature of the study.

## Results

Wilhelm et al [21] report the feasibility and acceptability of *Perspectives*, as well as the symptom improvement from baseline to posttreatment.

### Descriptive Patterns of App Usage

**Figure 1:**
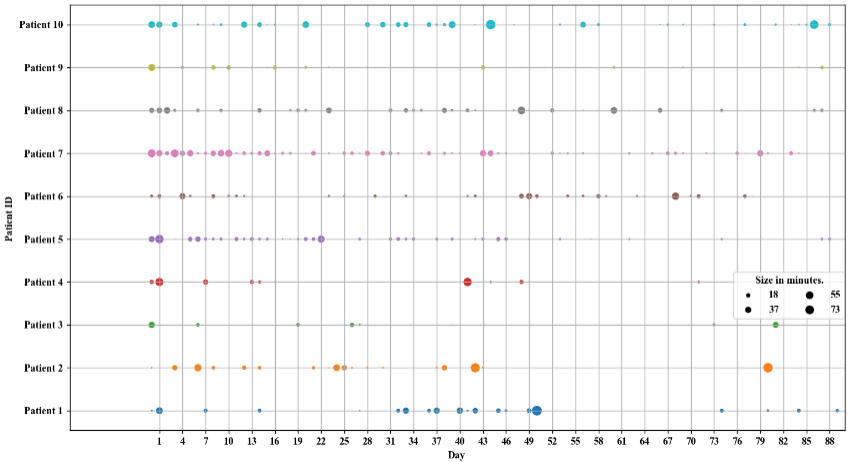
Individual patterns of engagement at a daily level. Usage is displayed as an aggregated sum of total minutes per day.

We visually examined the longitudinal patterns of engagement with *Perspectives* across the 12-week treatment (Figure 1). Overall, app usage showed a great deal of variety between participants, in terms of the total duration of use (mean duration 398 min, SD 310 min; range 53 to 913 min), the number of days used (mean 30 days, SD 16 days; range 8 to 64 days), and the length of time between consecutive app uses. This variety was also reflected in the qualitative descriptions of how participants used the app. Whereas some participants described using the app daily (eg, “in the evenings every day – I am not a morning person” and “when at my desk 30 minutes a day”), others engaged with it less frequently (eg, “usually once or several times a week”).

Despite the diversity in app usage across participants, several common usage patterns also emerged. First, most participants used the app with higher and lower intensities in the first and last weeks of the treatment, respectively (Figure 1). Additionally, data from both the app usage logs and GPS revealed that—within participants—participants generally preferred using the app at home over the first 8 weeks (1040/1488, 69.90% at home on average). During the ninth and tenth weeks, the proportion of app use at home and outside of home became more evenly distributed (60/105, 56.9% at home), and in the final 2 weeks of treatment, participants predominantly used the app outside of home (with only 16/98, 17% at home).

Moreover, unlike in-person therapy, most interactions with *Perspectives* were frequent (Figure 1) and very brief (Multimedia Appendix 1). The majority (374/510, 73.3%) of app sessions lasted ≤5 min. In only 11.7% (60/510) of cases, the app was used in sessions lasting 5 to 10 min, followed by 7.1% (36/510) and 6.4% (33/510) of cases in which the content was accessed for 10 to 20 or 20 to 40 min, respectively. Longer app usage was registered in only 1.4% (7/510) of the sessions. This pattern of brief engagement is consistent with how participants described their app usage in qualitative feedback. For instance, participants reported that they used the app during “dead time” while waiting (eg, in line at the store) or “for a few minutes each day to keep the lessons in mind” and described the app as “fast and easy to fit into your busy schedule.”

### App Usage Patterns as Correlates of Percentage Improvement in the Yale-Brown Obsessive Compulsive Scale Modified for Body Dysmorphic Disorder

Means, standard deviations, and bivariate correlations of the percentage improvement in the BDD-YBOCS, the quantity of app usage, and frequency of app usage are provided in Table 1.

**Table 1:** Descriptive statistics and correlations between patterns of engagement with smartphone-delivered cognitive behavioral therapy for body dysmorphic disorder and treatment response.

App usage patterns	Midtreatment, mean (SD)	Posttreatment, mean (SD)	Correlation with percentage improvement in BDD-YBOCS^a^ (baseline to week 12)^b^	*P* value
Percentage improvement in BDD-YBOCS (%)	32.3 (23.8)	45.3 (14.7)	N/A^c^	N/A
Quantity of app use	293.9 (276.4)	398.0 (310.25)	–0.084	.82
Frequency of app use^d^	6.24 (0.7)	6.44 (0.73)	–0.57	.08

^a^BDD-YBOCS: Yale-Brown obsessive compulsive scale modified for body dysmorphic disorder.

^b^Correlations calculated for quantity and frequency of app use were derived from full 12-week treatment.

^c^N/A: not applicable.

^d^Frequency of app use values are log-transformed. Nontransformed means at midtreatment and posttreatment are 512 min (SD 1 255 to 1032 min) and 626 min (SD 1 301 to 1299 min), respectively.

The quantity of app usage was uncorrelated with percentage improvement in the BDD-YBOCS, whereas the frequency of app usage correlated strongly with treatment response and trended toward significance. The strong, negative relationship between mean (log) length of breaks between sessions (ie, frequency of app use) and improvement in the BDD-YBOCS initially suggests that shorter breaks between sessions corresponded with greater improvements (Table 1).

To follow up on patterns elucidated in bivariate correlations, we used regression analysis to preliminarily examine whether the frequency of app usage corresponded with treatment response more so than the quantity of app usage. When the primary outcome (percentage improvement in the BDD-YBOCS) was entered as a dependent variable, the frequency of app usage (ie, mean (log) duration between 2 consecutive sessions) predicted percentage improvement in the BDD-YBOCS with a small effect (beta=–0.13; *P*=.03; 95% CI –0.231 to –0.019), whereas the total quantity of app usage during the 12-week treatment did not predict improvement in the BDD-YBOCS (beta=–0.08; *P*=.13; 95% CI –0.184 to 0.027).

### GPS Data as a Correlate of the Yale-Brown Obsessive Compulsive Scale Modified for Body Dysmorphic Disorder Scores

**Figure 2:**
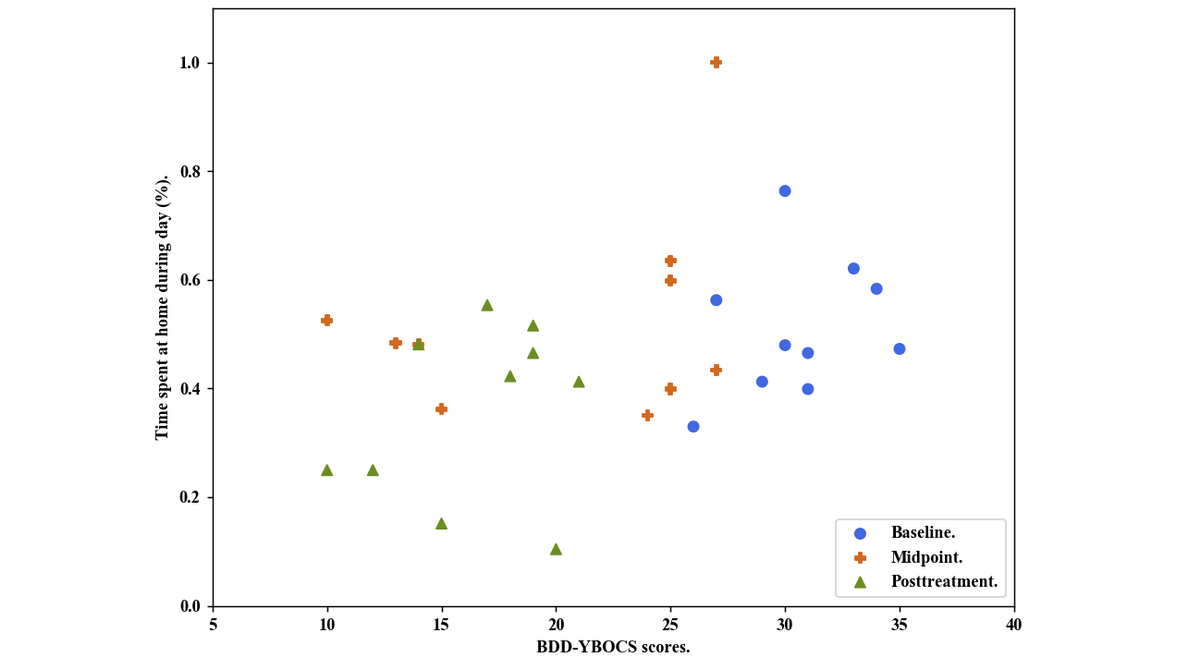
Body dysmorphic disorder severity and time spent at home across treatment. BDD-YBOCS: Yale-Brown obsessive compulsive scale modified for body dysmorphic disorder.

We used a scatterplot to visually inspect the relationship between time spent at home (based on GPS data) and symptom severity (measured with the BDD-YBOCS; Figure 2). The plot indicates that a shift occurred from baseline to posttreatment, characterized by a corresponding decrease in time spent at home and symptom severity. A follow-up correlational analysis suggests a moderately strong association between time spent at home and BDD symptom severity (*r*=0.49; *P*=.005).

## Discussion

### Principal Findings

Although enthusiasm for smartphone-delivered CBT is growing rapidly, there has not yet been substantial research on ways to enhance smartphone treatment. Before the widespread development and deployment of smartphone CBT treatments, it is important to first examine pilot data that characterizes the natural engagement patterns of users with smartphone-delivered CBT and identifies which usage and mobility patterns may optimize treatment. Such pilot data will provide timely information to researchers about variables and hypotheses of focus, in advance of larger, more costly validation trials, and can elucidate how we may explore enhancing smartphone-delivered CBT for optimum response in larger trials.

In particular, passively collected usage and sensor data from smartphones offer a unique, low-burden approach for gaining these important insights. Although a variety of passive data (eg, typing speed, activity level, phone usage, acoustic level) can be collected by smartphones [28], collecting sensor data involves a trade-off between gaining potentially useful information and depleting phone battery life (as well as risking user trust when collecting unnecessary data). Thus, initial signals from pilot research can shed light on which variables may be more or less fruitful to collect in clinical trials. To this end, this study used passive data from an open pilot trial of smartphone-delivered CBT for BDD, with the aim of preliminarily (1) characterizing the patterns of app usage of participants, (2) examining usage patterns that correspond with treatment response, and (3) examining mobility patterns that correspond with symptom severity.

Although app usage patterns varied substantially across participants, visual examination and descriptive analysis of usage data revealed several common patterns of engagement in our sample. First, participants tended to use the app more frequently and for a greater overall duration at the beginning of the 12-week treatment, with considerably lower usage later in treatment. This result is not surprising and may reflect that early on, participants required more time on the app to learn new information and skills. Later in the treatment, the participants may have transitioned to practicing greater applied skills, offline and in the real world [29]. In fact, qualitative feedback reflects that once participants learn skills, they practice them offline. For example, one participant reported, “I use the exercises all the time without the app. I have the big picture view of what I am trying to do.” Learning to use the treatment skills offline is likely an effective way to engage with smartphone-delivered CBT over time, as ultimately (like with in-person CBT), we hope for patients to internalize skills well enough to use them naturally as symptoms arise. Similarly, the results could reflect that participants simply received the necessary dose of treatment in a shorter time than the allotted 12 weeks [29]. On the other hand, lower usage at the end of treatment may reflect drops in engagement unrelated to CBT mastery (eg, because of boredom, lack of new content, loss of motivation). One participant’s posttreatment qualitative feedback supports this hypothesis; the participant reported that toward the end of the 12 weeks, there was less new material, and the participant was therefore not on the app as often. Reduced engagement over time is a very common challenge for app-based treatments [30]. Additional research is needed to fully understand the reasons for the reductions in app usage over time.

Second, descriptive results highlighted that participants typically used the app at home during the first two-thirds of treatment; later, the participants tended to use the app more when out of the house. This within-person pattern of increased usage outside of the house over time is consistent with the hypothesis that as participants gained CBT skills across treatment, they may have transitioned to using those skills offline and in the real world.

Finally, we observed that overall, the participants tended to use the app in brief and frequent sessions. In fact, most app sessions lasted <5 min each. This pattern reflects the way in which most people use smartphones in general: engaging with them often during short moments of downtime throughout the day [31]. This pattern also aligns with how we designed the app to be used. That is, we intentionally pared down content into brief text and exercises that could be completed quickly and repeated as often as one wished.

On the other hand, this pattern of brief and frequent sessions is notably distinct from how patients engage with face-to-face CBT or ICBT. Given the distinctive pattern of engagement we observed compared with better-established CBT modalities, it is critical to examine whether the naturally brief usage patterns of participants with smartphone-delivered CBT are effective or whether longer sessions are needed for response. Interestingly, preliminary correlation and regression results suggest that more frequent app usage, as opposed to greater duration of app usage, correlated strongly with treatment response—and trended toward statistical significance—in our (albeit small) sample. Consistent with these results, a previous review showed that overall time spent on web-based treatments for depression does not typically correlate with response to treatment [32]. In line with the aforementioned hypothesis that participants often practiced skills offline once learned, it is possible that the total duration of app usage does not fully capture the time participants spent engaging in treatment skills. Altogether, the results provide early, novel evidence that frequent (even if brief) practice of CBT skills may optimize the smartphone-delivered CBT response.

It is possible that frequent doses of practice help with learning CBT skills, as regular reinforcement of skills across broad contexts may enhance consolidation and generalization [33]. Researchers who are in the process of designing clinical trials to test smartphone-delivered CBT should consider collecting both quantity and frequency usage metrics to further validate optimum usage patterns. If validated in subsequent trials, the results have implications for the design of smartphone-delivered CBT. For example, findings suggest that information should be provided in brief chunks, as opposed to packing long, self-help–style psychoeducation into smartphone-delivered treatments. Moreover, it may be beneficial to design apps that are discreet, to promote frequent app use not only at home but also as symptoms arise in day-to-day life. App design can actively promote frequent use by incorporating reminders or rewards for use, in addition to including instructions to engage with the app often. Future research could test these design strategies using experimental designs to investigate which are effective for promoting frequent use.

In addition to usage patterns, we also examined mobility patterns from GPS data that correspond to BDD severity. Preliminary results showed that across treatment, the proportion of time spent at home—a potential digital marker of avoidance [17]—decreased. Time spent at home correlated positively with BDD severity across treatment, with a medium-to-large effect. Whether the proportion of time at home is truly tapping into avoidance behaviors (versus other aspects of BDD severity) is speculative and requires validation through future research. This is the first study to examine the time spent at home in relation to BDD severity. Whereas previous research has documented a link between time spent at home and depressive symptoms [18], because of the small sample, we did not examine this relationship when controlling for depression severity. However, as depression severity did not decrease across treatment in this sample [21], it is unlikely that the observed link is better accounted for by changes in depressive symptoms. Future research in a larger trial could parse apart the degree to which time spent at home serves as a digital marker of depressive versus BDD severity.

Altogether, strong initial GPS results underscore one variable where gains of data collection may outweigh costs; researchers designing upcoming smartphone-delivered CBT trials should consider measuring time spent at home, to further validate this potential unobtrusive marker of clinical severity. With further validation, detecting changes in one’s time spent at home could enhance smartphone-delivered CBT by unobtrusively triggering just-in-time interventions—a promising yet underdeveloped area of research [34]. For example, upon detecting increases in time spent at home, smartphone-delivered CBT treatments could send notifications to the user that reflect this observation (eg, “It looks like you’ve been spending more time at home”) and suggest adaptive strategies (eg, “Would you like to schedule an activity with a friend?”). Moreover, in larger trials, researchers can explore the utility of applying machine learning methods to predict changes in BDD severity from GPS-derived time spent at home.

### Limitations

Results from this study should be interpreted, bearing in mind its limitations. Most notably, this pilot study had a small sample size. Thus, it is possible for 1 or 2 participants’ outlying usage patterns to unduly influence the results. That said, Kazdin [35] outlines a strong rationale for the ability to meaningfully examine data from small samples when data are collected at multiple time points across the treatment. Given the small sample size, we limited the scope of our aims and analyses to an exploratory examination of select patterns of interest, and we focused on robust effects that may indicate meaningful signals to follow-up. Follow-up in a larger sample would provide an opportunity to reliably test for statistical significance. To this end, results are intended to hone researchers’ decisions (eg, variables and hypotheses of focus) in advance of larger, more costly clinical trials of smartphone-delivered CBT treatments rather than to provide conclusive evidence in and of themselves.

In addition to a small sample, this pilot trial specifically focused on smartphone-delivered CBT for BDD. It is possible that insights will not generalize to smartphone-delivered CBT treatments for other disorders. However, given the core similarities between CBT for BDD and many other psychiatric conditions, such as anxiety disorders, obsessive-compulsive–related disorders, and eating disorders, we anticipate that findings will be relevant in the design of smartphone-delivered CBT treatments for related conditions. Finally, our strong initial GPS results should be interpreted, bearing in mind the high degree of missing GPS data (683/1134, 60.23% of the days) in our sample. Although the specific reasons for missing GPS data in our study are unknown, a high rate of missing geolocation data in mobile research is typical (eg, ranging from 40% to 90% missing) [36-39] and may be attributed to a range of factors, including participants switching off the device, participants activating a mode that does not permit location services (eg, airplane mode) or problems with permission to access the location sensor that can occur with the iPhone platform [36]. Importantly, missing GPS data in our study did not correlate with the BDD symptom severity of the participants and therefore were likely random with respect to BDD symptoms. Thus, it is unlikely that patterns of missingness meaningfully influenced this correlation result. As with other results in this pilot study, these initial findings should be used for hypothesis generation at this stage.

### Conclusions

This study also had several notable strengths. First, whereas many existing smartphone-delivered CBT trials use nonclinical or convenience samples, we used a clinical sample that was diagnosed and assessed via gold-standard, clinician-administered measures. Participants were recruited nationally, which may enhance the generalizability of our initial findings. Finally, the correlation results for app usage and GPS patterns were robust despite our small sample, suggesting that these novel insights have strong potential to enhance costly, well-powered future trials.

Altogether, the results suggest that as researchers design efficacy trials to test smartphone-delivered CBT, it is worthwhile to collect data on patterns of use (with a focus on frequency versus quantity of use) and time spent at home. Novel study results suggest that these variables may correspond meaningfully with the response to treatment and, with further validation, may inform how to enhance smartphone-delivered CBT interventions.

## Appendix

Multimedia Appendix 1 Distribution of session durations aggregated across the sample.

## Abbreviations

BDD: body dysmorphic disorder
BDD-YBOCS: Yale-Brown obsessive compulsive scale modified for body dysmorphic disorder
CBT: cognitive behavioral therapy
HIPAA: Health Insurance Portability and Accountability Act
ICBT: internet-based cognitive behavioral therapy
