# Supplementary figures and images for: Optimizing Smartphone-Delivered Cognitive Behavioral Therapy for Body Dysmorphic Disorder Using Passive Smartphone Data: Initial Insights From an Open Pilot Trial
Source: JMIR Mhealth Uhealth. 2020 Jun 18;8(6):e16350. doi: 10.2196/16350 (PMC7333068; doi:10.2196/16350)

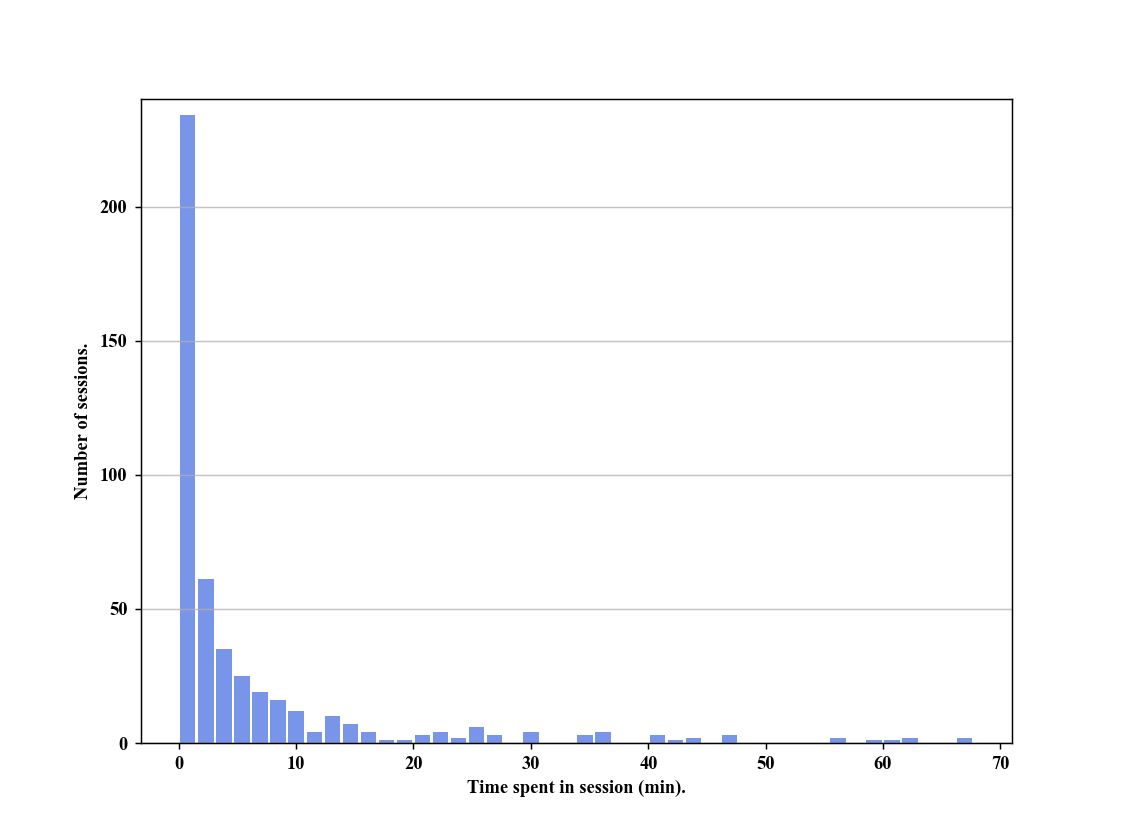

Supplement: Multimedia Appendix 1 [file mhealth_v8i6e16350_app1.png]
